# Association of systemic inflammation with balance and falls in older adults: National Health and Nutrition Examination Survey and Mendelian randomization study

**DOI:** 10.1093/gerona/glaf242

**Published:** 2025-10-31

**Authors:** Sodiq Fakorede, Kai Cheng, Olubodun M Lateef, Okikiola Samuel Fakorede, Deqiang Wang, Dan Wang, Xia Liu

**Affiliations:** Department of Physical Therapy, Rehabilitation Science, and Athletic Training, University of Kansas Medical Center, Kansas City, Kansas, United States; School of Special Education and Rehabilitation, Binzhou Medical University, Yantai, China; Department of Medical Pharmacology and Physiology, University of Missouri Columbia, Columbia, Missouri, United States; Department of Physiotherapy, University of Ibadan, Ibadan, Nigeria; Department of Rehabilitation Medicine, Yantai Affiliated Hospital of Binzhou Medical University, Yantai, China; Department of Rehabilitation Medicine, Yantai Affiliated Hospital of Binzhou Medical University, Yantai, China; Department of Rehabilitation Medicine, Yantai Affiliated Hospital of Binzhou Medical University, Yantai, China; (Medical Sciences Section)

**Keywords:** C-reactive protein, Balance performance, Fall risk, Mendelian randomization

## Abstract

**Background:**

Falls are a leading cause of morbidity in older adults, with emerging evidence suggesting that systemic inflammation may contribute to this risk. C-reactive protein (CRP), a biomarker of inflammation, has been linked to various health issues, including declines in physical function. However, its direct influence on balance and fall risk remains uncertain. This study investigates the association between CRP levels and balance using observational data and Mendelian randomization (MR) to explore its causal role in fall risk.

**Methods:**

We analyzed data from the 2021-2023 National Health and Nutrition Examination Survey (NHANES), including 1215 participants aged 60 and older. CRP levels were measured using immunoturbidimetric assays, and balance was assessed via the Modified Romberg Test. We used multivariable ordinal logistic regression models to evaluate the relationship between CRP and balance, adjusting for demographic, health, and lifestyle factors. Genetic instruments for CRP were derived from genome-wide association studies (GWAS), and MR analysis was performed using fall risk summary statistics (2215 cases, 6289 controls).

**Results:**

In the NHANES cohort, higher CRP levels were associated with poorer balance (*β* = −0.201, *p* = .007). This association was stronger in males but not in females. MR analysis confirmed a causal link between elevated CRP and increased fall risk (OR = 1.13, *p* = 8.96 × 10^−8^), with no evidence of pleiotropy or heterogeneity.

**Conclusions:**

our findings highlight CRP as a key factor influencing balance and a causal contributor to fall risk in older adults, suggesting that anti-inflammatory interventions may help reduce fall risk.

## Introduction

The global demographic shift toward an aging population has intensified the need to address balance impairments and fall-related injuries in older adults.^1^^,^^2^ Falls are a leading cause of morbidity and mortality in this age group, placing a significant burden on individuals, families, healthcare systems, and society.^3^ Balance performance is a key determinant of fall risk and is essential for maintaining mobility, independence, and overall quality of life.^4^^,^^5^ Impaired balance often leads to a cycle of reduced physical activity, increased frailty, and loss of autonomy, making the prevention of balance dysfunction a critical public health priority.^2^^,^^6^

Systemic inflammation has emerged as a potential contributor to balance impairments and fall risk.^7^ Chronic low-grade inflammation is linked to conditions that negatively affect balance, including sarcopenia, neurodegenerative disorders, and frailty.^8^ C-reactive protein (CRP), a widely recognized biomarker of systemic inflammation, is produced by the liver in response to pro-inflammatory cytokines and serves as a reliable indicator of inflammatory status.^9^^,^^10^ Elevated CRP levels have been associated with adverse health outcomes such as cardiovascular disease, diabetes, and physical function decline, underscoring its relevance in understanding the mechanisms underlying balance impairments.^11^^,^^12^

Although systemic inflammation has been linked to physical function,^11^^,^^13^ the specific relationship between CRP levels and balance performance remains inadequately explored. Observational studies have reported conflicting findings—some identify a significant association between elevated CRP levels and balance impairments,^14^ while others find no such relationship after adjusting for confounders.^12^ These inconsistencies may stem from inherent limitations in observational research, including confounding factors such as age, comorbidities, and lifestyle behaviors, as well as the possibility of reverse causation.^15–17^ As a result, it remains unclear whether elevated CRP levels directly contribute to dysfunction of balance or simply reflect underlying health conditions.

Mendelian Randomization (MR) provides a powerful tool to clarify this uncertainty.^18^^,^^19^ By using genetic variants associated with CRP levels as instrumental variables (IVs), MR analysis can infer causal relationships while reducing bias from confounding and reverse causation.^20^ Recent genome-wide association studies (GWAS) have identified strong genetic instruments for CRP, enabling the application of MR methodologies. Additionally, GWAS datasets on fall risk, balance performance, and mobility offer a valuable opportunity to investigate the causal role of systemic inflammation in fall susceptibility.

This study combines observational and genetic approaches to provide a comprehensive analysis of the relationship between CRP and balance performance. First, we leverage data from the National Health and Nutrition Examination Survey (NHANES), a population-based dataset rich in demographic, health, and biomarker information, to examine the association between CRP levels and balance performance. Second, we apply MR using summary statistics from GWAS for CRP and fall risk to assess whether systemic inflammation causally influences balance-related outcomes. This integrated approach addresses the limitations of traditional observational studies and offers insights into the biological mechanisms contributing to fall risk. By combining NHANES observational data with MR analysis, this study aims to clarify the role of systemic inflammation in balance performance and its implications for fall risk. Should a causal link between CRP levels and balance dysfunction be confirmed, it could guide the development of targeted anti-inflammatory interventions to enhance balance and reduce fall risk in aging populations.

## Methods

### Data sources

We obtained cross-sectional data from the 2021-2023 cycle of the NHANES, a population-based program conducted by the National Center for Health Statistics (NCHS). The NCHS Ethics Review Committee approved NHANES, and all participants provided written informed consent before enrollment. The NCHS Research Ethics Review Board authorized the study under protocol #2021-05. NHANES follows strict protocols to ensure confidentiality and protect against identification. Since this analysis utilized publicly available data, additional institutional review board approval was not required. The study adhered to the STROBE (Strengthening the Reporting of Observational Studies in Epidemiology) guidelines to ensure transparent and rigorous reporting.^21^^,^^22^ The NHANES dataset is accessible through the official website (https://wwwn.cdc.gov/nchs/nhanes/continuousnhanes/default.aspx? Cycle=2021-2023).

### Participants and study design

**Figure 1.**
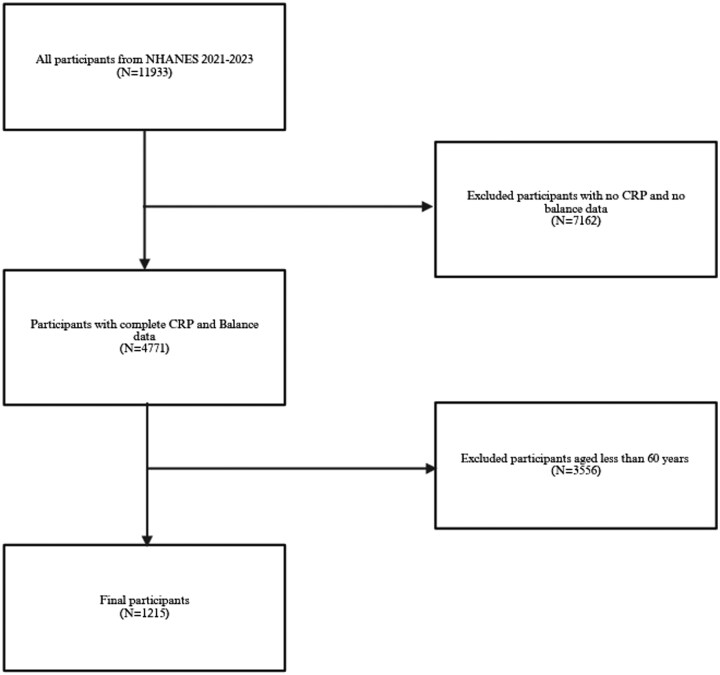
Inclusion and exclusion criteria.

NHANES, a nationally representative health survey led by the NCHS under the Centers for Disease Control and Prevention (CDC), provides critical health statistics for the U.S. population. For this study, we analyzed cross-sectional data from the NHANES 2021-2023 cycle, which included 8727 participants with CRP measurements and 4771 participants with balance assessments. To focus on older adults, we restricted the analysis to individuals aged ≥60 years. This threshold is consistent with the definition of an older person used by the United Nations^23^ and other aging research.^24^^,^^25^ Participants with missing serum CRP levels and/or balance performance data were excluded, yielding a final study sample of 1215 participants (Figure 1).

### C-reactive protein

We extracted CRP data from the NHANES 2021-2023 dataset. Blood samples were collected, processed, and stored before being shipped to the University of Minnesota’s Advanced Research Diagnostics Laboratory (ARDL) in Minneapolis, MN, for analysis. CRP concentrations were measured using an immunoturbidimetric assay, which detects CRP by aggregating latex particles coated with anti-CRP antibodies. Initially, blood samples were mixed with a Tris buffer, followed by the addition of latex particles. In the presence of CRP, these particles formed immune complexes, increasing turbidity. The degree of turbidity, which correlates with CRP concentration, was measured and compared against a standard calibration curve. CRP levels were quantified using a Cobas 8000 Analyzer (Roche Diagnostics, Indianapolis, IN), with absorbance readings taken at a primary wavelength of 546 nm and a secondary wavelength of 800 nm. This highly sensitive assay allowed for precise CRP measurement, making it suitable for detecting systemic inflammation in individuals without acute inflammatory conditions. Laboratory analyses followed NHANES quality control protocols and complied with the Clinical Laboratory Improvement Amendments (CLIA) of 1988 to ensure accuracy and reliability.

### Assessment of balance

Balance assessment was based on the Modified Romberg Test (MRT) from the NHANES 2021-2023 dataset.^26^ Participants wore a gait belt for safety and were tested under five progressively challenging conditions. These included standing with eyes open on a firm surface (Condition 1), standing with eyes closed on a firm surface (Condition 2), standing with eyes open on a foam surface (Condition 3), standing with eyes closed on a foam surface (Condition 4), and standing on foam with eyes closed while moving the head side-to-side (Condition 5). For all conditions, participants were instructed to stand with their feet together and arms crossed. A test was considered passed if balance was maintained for the designated time: 15 s for Conditions 1 and 2, and 20 s for Conditions 3, 4, and 5. Failure occurred if participants moved their feet or arms, opened their eyes during eyes-closed conditions, touched the wall, or required assistance to prevent a fall. This study focused on pass/fail outcomes rather than the exact duration of time spent in each condition.

No specific cut-off point was applied to define “balance impairment”; instead, the ordinal score was used to model the continuum of postural control ability. While MRT does not directly measure fall risk, prior research has demonstrated that poor performance on balance assessments such as the MRT is associated with increased fall risk and functional decline in older adults.^4^^,^^26^ Therefore, MRT performance was used as a proxy indicator of balance performance, which itself is a known contributor to fall risk.^27^

## Statistical analysis

Statistical analyses were performed using R Statistical Software (version 4.4.2) (R Foundation for Statistical Computing, Vienna, Austria). We accounted for the NHANES dataset’s complex, multistage probabilistic sampling design using the survey package, which adjusted for stratification, clustering, and sample weights to ensure nationally representative estimates.^28^ Missing data for the covariates were imputed with the mice package, and the imputed dataset was used for all analyses. Descriptive statistics were calculated for all variables. Continuous variables were summarized using means and standard deviations, with gender differences assessed via *t*-tests. Categorical variables were expressed as percentages, and group differences were evaluated with chi-square tests.

### Ordinal logistic regression

To correct data skewness, CRP values were log-transformed. We explored the relationship between log-transformed CRP and postural balance performance using three ordinal logistic regression models. Postural balance performance was treated as an ordinal variable, representing the number of MRT conditions successfully completed. Model 1 examined the unadjusted association between log-transformed CRP and balance performance. Model 2 adjusted for key demographic and socioeconomic factors, including age, gender, race/ethnicity, and education level. Model 3 further adjusted for health and lifestyle factors, such as body mass index, family income-to-poverty ratio, diabetes status, blood pressure, cholesterol levels, physical activity, smoking status, and alcohol consumption. These potential confounders were selected a priori based on established evidence from previous literature suggesting their association with both systemic inflammation and balance control in older adults.^29^^,^^30^ This comprehensive adjustment aimed to control for potential confounders that could influence the relationship between CRP and balance performance.

To assess potential gender-specific differences, we conducted subgroup analyses by fitting separate ordinal logistic regression models (Models 1-3) for males and females. While the overall models were adjusted for gender as a covariate, these subgroup analyses used gender as a stratification variable; therefore, gender was not included as a covariate within the separate models for males and females. This approach allowed for the comparison of regression coefficients across genders and the identification of any differences in the association between CRP and postural balance. All results are presented as regression coefficients (estimates) with 95% confidence intervals and *p*-values. Statistical significance was set at *p* < .05 for all tests.

### Mendelian randomization study

**Figure 2.**
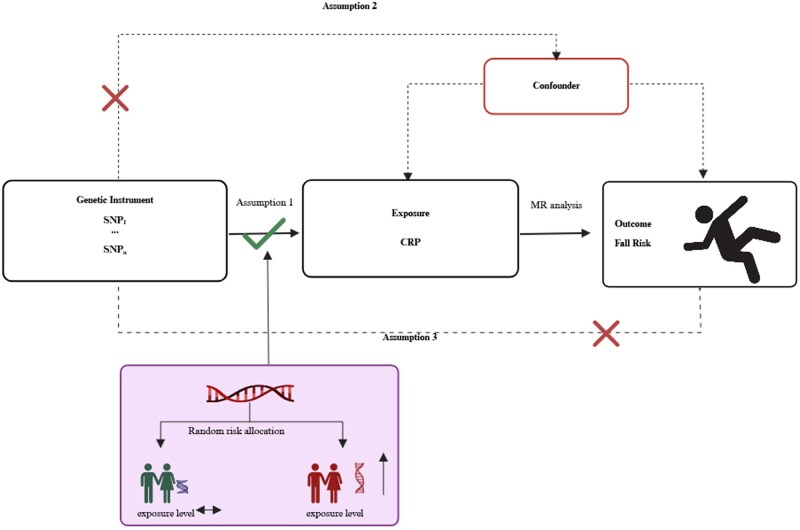
Mendelian randomization assumptions and principles.

To ensure the validity of our MR analysis exploring the causal link between CRP levels and fall risk (balance impairment), we followed established triangulation principles (Figure 2).^31^ Specifically, we required: (i) strong and consistent associations between the selected genetic variants and CRP levels (the exposure); (ii) independence of these variants from potential confounding factors to prevent spurious associations; and (iii) that the genetic variants affect fall risk solely through their influence on CRP levels, upholding the exclusion restriction assumption.^31^ Our approach utilized single nucleotide polymorphisms (SNPs) associated with CRP levels as IVs, identified through GWAS data, which provides large-scale, high-quality genetic information. This methodology reduces biases commonly encountered in traditional observational studies, enhancing the reliability of our conclusions regarding the causal relationship between CRP levels and fall risk.

#### Selection of genetic instruments

We extracted genome-wide significant SNPs (*p* < 5 × 10−8 associated with serum CRP levels from the UK Biobank summary statistics via the IEU OpenGWAS database (https://gwas.mrcieu.ac.uk/datasets/ukb-d-30710_irnt/). The UK Biobank represents one of the most comprehensive datasets available, encompassing data from 343 524 participants for CRP analysis. We selected IVs through a rigorous quality control process, systematically identifying genetic variants that satisfied the core assumptions of MR analysis.^32^ To ensure the independence of genetic instruments and avoid linkage disequilibrium (LD), we performed clumping using PLINK. SNPs with an *r*^2^ > 0.001 within a 10 000 kb window were excluded. Additionally, palindromic SNPs with intermediate allele frequencies were removed to prevent potential biases. We calculated the *F*-statistic (*F*_exposure = $\frac{\mathrm{Beta}\_\mathrm{exposure}2\;}{\mathrm{SE}\_\mathrm{exposure}2}$) for each variant to assess IV strength and validity, retaining only those with an *F*-statistic greater than 10.^33^ All effect alleles were harmonized to align with the CRP-increasing allele. A detailed list of the SNPs used as IVs is provided in Table S1. For fall risk, we sourced GWAS summary-level data from the GWAS Catalog (https://www.ebi.ac.uk/gwas/rest/api/studies/GCST90012857), which included 2215 cases and 6289 controls, all of European ancestry. This outcome was selected as the most clinically relevant proxy for severe balance impairment due to the current lack of publicly available, large-scale GWAS summary data for direct measures of balance performance.

#### MR analysis

We primarily employed the IVW method, which is known for providing unbiased estimates under the assumption of no horizontal pleiotropy. IVW was the main tool used to infer causality between CRP levels and fall risk. To assess variability among the genetic instruments,^34^ we conducted the Cochrane *Q* heterogeneity test. To further validate our findings and examine potential violations of instrumental variable assumptions, we applied additional MR methods, including the Weighted Median, Simple Mode, and Weighted Mode approaches.^35^^,^^36^ The Weighted Median method is particularly robust, offering consistent estimates even when up to 50% of the instruments are invalid due to horizontal pleiotropy. The Simple and Weighted Mode methods, on the other hand, rely on subsets of instruments with consistent causal effects to generate reliable estimates.

We assessed the potential for horizontal pleiotropy using the MR-Egger regression intercept, which tests for significant deviation from zero.^37^ A nonsignificant intercept suggests no substantial directional pleiotropy. Additionally, we applied the MR-PRESSO global test to identify and correct for any pleiotropic outlier variants. To further ensure the robustness of our results, we conducted sensitivity analyses, including a leave-one-out analysis, where we systematically removed each SNP and recalculated the causal effect. This analysis ensured that no single SNP disproportionately influenced the observed causal effect.

We set statistical significance at *p* < .05 for all tests. Results were presented as odds ratios (ORs) with corresponding standard errors (SEs) and 95% confidence intervals (CIs). All analyses were performed using the TwoSampleMR package and the MR-PRESSO framework in R software (version 4.2.3). These methods contributed to the reliability and robustness of the causal inferences drawn in this study.

## Results

### Study participant demographics

Table 1 summarizes the demographic and clinical characteristics of the study participants (*n *= 1215), including 545 males and 670 females. The average age of participants was 64.32 ± 2.86 years, with no significant age differences between genders. However, significant gender differences were observed in body mass index (BMI), total cholesterol levels, and log-transformed CRP levels.

**Table 1.** Baseline characteristics of the study population by gender.

Variable	Male (*n* = 545)	Female (*n* = 670)	Total (*n* = 1215)	*p*-Value
**Age (years)**	64.32 ± 2.89	64.33 ± 2.85	64.32 ± 2.86	.946
**BMI (kg/m²)**	28.90 ± 5.22	29.59 ± 6.64	29.28 ± 6.05	.043
**Total cholesterol (mg/dL)**	182.80 ± 41.26	203.17 ± 42.65	194.04 ± 43.22	<.001*
**CRP (log)**	1.10 ± 0.70	1.23 ± 0.70	1.17 ± 0.70	.001*
**Poverty-income ratio**	3.20 ± 1.67	3.15 ± 1.67	3.17 ± 1.67	.627
**Race/Ethnicity (%):**				.448
** Mexican American**	5	4.3	4.6	
** Other Hispanic**	8.1	8.2	8.1	
** Non-Hispanic White**	64	67	65.7	
** Non-Hispanic Black**	12.5	10.6	11.4	
** Asian**	4.2	5.5	4.9	
** Multi-racial**	6.2	4.3	5.2	
**Education level (%):**				.05
** Less than 9th grade**	5.0	5.5	5.3	
** 9th-11th grade**	9.2	5.4	7.1	
** High school graduate**	24.6	21.5	22.9	
** Some college**	30.5	33.1	31.9	
** College graduate or above**	30.8	34.5	32.8	
**High blood pressure (%):**				.099
** Yes**	51.2	46.3	48.5	
** No**	48.8	53.7	51.5	
**Smoking status (%):**				.89
** Everyday**	23.1	24.2	23.7	
** Someday**	6.1	6.3	6.2	
** Not at all**	70.8	69.6	70.1	
**MRT score**				.8017
** 0**	0.7	0.3	0.5	
** 1**	1.5	1.8	1.6	
** 2**	2.2	1.9	2.1	
** 3**	33.9	32.5	33.2	
** 4**	36.3	35.7	36	
** 5**	25.3	27.8	26.7	
**Diabetes status (%):**				.151
** Yes**	19.3	15.1	17.0	
** No**	76.0	79.7	78.0	
** Borderline**	4.8	5.2	5.0	
**Alcohol use (%):**				<.001*
** Never last year**	18.3	22.1	20.4	
** 1-2 times/year**	7.2	14.0	10.9	
** 3-6 times/year**	5.9	12.2	9.4	
** 7-11 times/year**	5.1	5.8	5.5	
** Once/month**	6.8	4.9	5.8	
** 2-3 times/month**	9.2	8.7	8.9	
** Once/week**	7.7	6.9	7.2	
** 2 times/week**	12.8	8.7	10.5	
** 3-4 times/week**	9.5	9.4	9.5	
** Nearly every day**	9.9	4.3	6.8	
** Every day**	7.5	3.0	5.0	
**Physical activity (min)**	120.62 ± 737.75	115.34 ± 767.94	117.71 ± 754.24	.903
**Coronary heart disease (%):**				<.001*
** Yes**	9.9	3.4	6.3	
** No**	89.2	96.6	93.3	
** Don’t know**	0.9	0.0	0.4	

Abbreviations: BMI: body mass index; CRP: C-reactive protein. MRT: Modified Romberg Test.

* *p* < .05.

### Association between CRP and performance of balance

The relationship between log-transformed CRP and postural balance performance was evaluated across three models, with separate subgroup analyses for males and females (Table 2). In Model 1, which examined the unadjusted association, CRP was significantly associated with postural balance performance in the overall sample (*β* = −.273; 95% CI: −.390, −.156; *p* < .001) and among males (*β* = −.364; 95% CI: −.605, −.124; *p* = .003). However, the association was not significant in females (*β* = −.205; 95% CI: −.434, 0.024; *p* = .079). In Model 2, after adjusting for demographic and socioeconomic factors, the association remained significant in the overall sample (*β* = −.226; 95% CI: −.354, −.097; *p* < .001) and among males (*β* = −.316; 95% CI: −.544, −.087; *p* = .007). For females, the association remained non-significant (*β* = −.160; 95% CI: −.383, 0.063; *p* = .160). Model 3 further adjusted for health and lifestyle factors. The association remained significant in the overall sample (*β* = −.201; 95% CI: −.349, −.054; *p* = .007) and among males (*β* = −.352; 95% CI: −.677, −.028; *p *= 0.033). In contrast, the association remained nonsignificant in females (*β* = −.063; 95% CI: −.326, 0.199; *p* = .636).

**Table 2.** Association between CRP level and balance performance.

Model	Group	Estimate (*β*)	Lower CI	Upper CI	*p*-Value
**Model 1**	Overall	−0.273	−0.39	−0.156	<.0001*
	Males	−0.364	−0.605	−0.124	.00297*
	Females	−0.205	−0.434	0.024	.0794
**Model 2**	Overall	−0.226	−0.354	−0.097	<.0001*
	Males	−0.316	−−0.544	−0.087	.00671*
	Females	−0.16	−0.383	0.063	.16
**Model 3**	Overall	−0.201	−0.349	−0.054	.00736*
	Males	−0.352	−0.677	−0.028	.0333*
	Females	−0.063	−0.326	0.199	.636

Abbreviations: CRP: C-reactive protein; CI: confidence interval.

Model 1 assessed the unadjusted association between log-transformed CRP levels and postural balance performance. Model 2 adjusted for key demographic and socioeconomic covariates, including age, gender, race/ethnicity, and education level. Model 3 further adjusted for additional health and lifestyle factors, including body mass index, family income-to-poverty ratio, diabetes status, blood pressure medication use, total cholesterol levels, physical activity, smoking status, and alcohol use.

* *p* < .05.

### Mendelian randomization analysis

A total of 92 SNPs associated with CRP levels were selected as genetic instruments. The *F*-statistics for these SNPs ranged from 21.78 to 529 (Table S1), indicating strong instrument strength and reducing the risk of weak instrument bias.

The primary MR analysis performed using the IVW method (Table 3), demonstrated a significant association between CRP levels and fall risk (OR = 1.13, 95% CI: 1.08-1.19, *p* < .001). Sensitivity analyses supported this finding. The Weighted Median method produced consistent results (OR = 1.11, 95% CI: 1.04-1.18, *p =* .003). However, both the Weighted Mode (OR = 1.08, 95% CI: 0.96-1.21, *p* = .217) and Simple Mode (OR = 1.06, 95% CI: 0.91-1.23, *p* = .449) methods did not reveal significant associations. The MR-Egger method also produced a non-significant causal estimate (OR = 1.07, 95% CI: 0.96-1.19, *p* = .229), with its intercept showing no evidence of directional pleiotropy (*p* = .250). Cochran’s *Q* statistic indicated no significant heterogeneity among the genetic instruments for both the IVW method (*Q* = 63.44, *p* = .988) and the MR-Egger method (*Q* = 62.10, *p* = .989). Additionally, the MR-PRESSO global test detected no significant outliers (*p* = .990). Finally, a leave-one-out sensitivity analysis confirmed the robustness of our findings, as the exclusion of any single SNP did not substantially affect the results (Figure S1, see online supplementary material for a color version of this figure). Collectively, these findings provide strong evidence supporting a causal link between systemic inflammation, as measured by CRP, and increased fall risk (Figure S2, see online supplementary material for a color version of this figure).

**Table 3.** Mendelian randomization analysis results.

Method	MR		Heterogeneity		Pleiotropy
	(OR [95% CI])	*p-Value*	*Q*	*p-Value*	Egger intercept
**Inverse variance weighted**	1.13 (1.08, 1.19)	8.96 × 10^-08*^	63.44	.9876	0.0015
**Weighted median**	1.11 (1.04, 1.18)	.0029*			
**Simple mode**	1.06 (0.91, 1.23)	.4487			
**Weighted mode**	1.08 (0.96, 1.21)	.2168			
**MR-Egger**	1.07 (0.96, 1.19)	.2294	62.1	.9891	

Abbreviations: OR: odds ratio; CI: confidence interval.

* *p* < .05.

## Discussion

This study explored the relationship between systemic inflammation, as indicated by CRP levels, and balance performance. We used data from NHANES for association analysis and GWAS data for MR analysis. Our analysis of the NHANES data revealed a significant link between elevated CRP levels and poor balance performance in the overall sample, with a particularly strong association observed in males. This relationship remained significant across increasingly adjusted models, supporting its robustness and independence from demographic, socioeconomic, health, and lifestyle factors. However, no significant association was found in females, suggesting potential gender differences in the effects of systemic inflammation on balance. In the MR analysis, which aimed to examine the causal effect of CRP on fall risk, the primary IVW method provided compelling evidence for a potential causal relationship. Sensitivity analyses using the Weighted Median method confirmed these results. Additionally, no significant evidence of pleiotropy or heterogeneity was found, further reinforcing the validity of the MR analysis. The findings from our NHANES analysis suggest a plausible mechanism for this causal link: higher CRP is associated with poorer balance, which is a primary risk factor for falls. Together, these findings suggest that systemic inflammation, as reflected by CRP levels, may have a causal role in balance performance, particularly in males, and could be a promising target for interventions to reduce fall risk and support healthy aging.

This study adds to the growing evidence linking systemic inflammation to physical function and balance performance.^11^^,^^38^ Previous research has highlighted the detrimental effects of elevated CRP levels on various aspects of physical performance, including gait speed, grip strength, and frailty.^12^^,^^38^^,^^39^ Our NHANES analysis supports these findings, showing a strong association between CRP levels and impaired balance performance, especially in males, across multiple models that accounted for demographic, socioeconomic, health, and lifestyle factors.

The gender differences observed in our study deserve special attention. Research suggests that systemic inflammation, as measured by CRP, may have a more pronounced impact on physical function in males.^40–42^ This aligns with prior studies that found CRP levels negatively correlate with muscle strength and physical performance in older males, but not females.^40^^,^^41^ Sex-specific differences in inflammatory responses, along with the protective effects of estrogen, may explain these differences. While females generally have higher baseline CRP levels, estrogen’s anti-inflammatory and neuroprotective properties may counteract the negative effects of inflammation on muscle function and neuromuscular control.^43^^,^^44^ Additionally, females may rely more on sensory and cognitive strategies to maintain balance, which reduces their reliance on muscle strength, the aspect most directly influenced by inflammation.^45^ In contrast, males, who are more dependent on muscle strength and neuromuscular coordination, may be more vulnerable to the impairments caused by inflammation, resulting in a stronger association between CRP levels and balance performance. These findings highlight the need to consider sex-specific mechanisms when studying the relationship between inflammation and physical function in older adults.

Unlike previous studies that relied exclusively on observational data,^7^^,^^12^ our inclusion of MR analysis enhances the causal inference regarding CRP’s impact on balance performance. The MR results, which reveal a significant causal relationship between systemic inflammation and balance-related outcomes using the IVW method, support emerging evidence suggesting that inflammation may not be a mere byproduct but rather a potential upstream factor contributing to balance impairment and falls.^46^ The consistency of our MR findings with sensitivity analyses further reinforces the robustness of this causal link.

However, the lack of a causal relationship observed in some sensitivity analyses, such as those using the Weighted Mode and MR-Egger methods, underscores the complexity of systemic inflammation’s effect on balance performance. This variation aligns with previous MR studies investigating the impact of CRP on other health outcomes, such as cardiovascular disease and frailty, where causal estimates have similarly varied across different analytical approaches.^47^^,^^48^ These discrepancies may reflect heterogeneity in the pathways linking CRP to balance performance or limitations in the genetic instruments used in MR studies.

Our study has several limitations. First, the generalizability of our findings may be constrained. Although NHANES represents the U.S. population, the genetic data used in our MR analysis is derived primarily from individuals of European ancestry. As a result, these findings may not fully apply to populations with different genetic backgrounds, lifestyles, or healthcare systems. Additionally, the NHANES dataset’s diverse, multiracial composition may introduce variability in the observational results, as genetic predispositions and environmental factors can influence balance performance.

Another limitation is the cross-sectional design of the NHANES data, which prevents us from establishing causal or temporal relationships between CRP levels and balance performance. Moreover, while the MRT used to assess balance performance is practical, we acknowledge that balance impairment and fall risk are related but distinct constructs. The ordinal MRT score models gradations in postural control, but does not directly quantify fall events. Despite adjusting for numerous potential confounders, unmeasured factors, such as diet, medication use, other inflammatory markers, or muscle strength, may still influence the observed associations. Notably, grip strength or other direct measures of muscular performance were not available in the 2021-2023 NHANES cycle, limiting our ability to account for the influence of muscular strength on balance. Additionally, the single-time point CRP measurements in NHANES do not capture potential short-term fluctuations or the effects of chronic inflammation over time. Finally, our study focused exclusively on CRP as a biomarker of systemic inflammation. While CRP is a robust and clinically relevant marker, the inclusion of other pro-inflammatory cytokines, such as Interleukin-6 or Tumor Necrosis Factor-alpha, would have provided a more complete assessment of the inflammatory pathways involved. The unavailability of these markers in the recent NHANES public-use dataset precluded such an analysis.

In conclusion, this study identifies a significant association between elevated CRP levels and impaired balance performance, as well as a causal relationship between CRP and increased fall risk. By integrating NHANES observational data with MR analysis, we provide strong evidence supporting the role of systemic inflammation in balance dysfunction and fall risk in older adults. Targeting chronic inflammation may be a promising strategy to improve balance and reduce fall risk. Future research should aim to validate these findings and explore effective interventions.

## Supplementary Material

glaf242_Supplementary_Data

## Data Availability

The datasets analyzed during the current study are publicly available. The observational data from the National Health and Nutrition Examination Survey (NHANES) 2021–2023 cycle are available from the Centers for Disease Control and Prevention (CDC) website at https://wwwn.cdc.gov/nchs/nhanes/continuousnhanes/default.aspx?Cycle=2021-2023. For the Mendelian Randomization analysis, the GWAS summary statistics for C-reactive protein (CRP) were obtained from the UK Biobank via the IEU OpenGWAS database (Dataset ID: ukb-d-30710_irnt) at https://gwas.mrcieu.ac.uk/datasets/ukb-d-30710_irnt/. The GWAS summary statistics for fall risk were obtained from the GWAS Catalog (Study Accession: GCST90012857) at https://www.ebi.ac.uk/gwas/rest/api/studies/GCST90012857.
