# Supplementary material for: Association of systemic inflammation with balance and falls in older adults: National Health and Nutrition Examination Survey and Mendelian randomization study
Source: J Gerontol A Biol Sci Med Sci. 2025 Oct 31;81(1):glaf242. doi: 10.1093/gerona/glaf242 (PMC12758964; doi:10.1093/gerona/glaf242)
Supplement: glaf242_Supplementary_Data [file glaf242_supplementary_data.pdf]

Captions

eTable 1: Genetic Instruments

eFigure 1: Leave-one-out Analysis

eFigure 2: Scatter plot of the SNP-CRP associations against SNP-fall risk associations

## eTable 1: Genetic Instruments

| SNP | Effect Allele | Other Allele | Beta | EAF | SE | P-value | F-statistic |
|---|---|---|---|---|---|---|---|
| rs10760691 | G | A | 0.017 | 0.383 | 0.003 | 8.90E-12 | 32.11111 |
| rs10831676 | A | C | 0.015 | 0.539 | 0.002 | 8.39E-10 | 56.25 |
| rs11145763 | C | A | 0.018 | 0.433 | 0.002 | 4.16E-14 | 81 |
| rs114414824 | T | C | 0.036 | 0.959 | 0.006 | 4.01E-09 | 36 |
| rs11577023 | T | C | 0.016 | 0.69 | 0.003 | 6.12E-10 | 28.44444 |
| rs115785198 | T | C | 0.051 | 0.071 | 0.005 | 1.60E-27 | 104.04 |
| rs11617494 | A | G | 0.016 | 0.241 | 0.003 | 2.23E-08 | 28.44444 |
| rs11693537 | A | G | 0.014 | 0.419 | 0.002 | 4.21E-09 | 49 |
| rs117211511 | A | G | 0.036 | 0.044 | 0.006 | 1.07E-09 | 36 |
| rs11777625 | C | T | 0.022 | 0.546 | 0.002 | 8.48E-21 | 121 |
| rs11850396 | G | A | 0.016 | 0.64 | 0.002 | 7.13E-11 | 64 |
| rs12132412 | G | A | 0.016 | 0.388 | 0.002 | 3.38E-11 | 64 |
| rs1223801 | G | A | 0.02 | 0.163 | 0.003 | 1.11E-09 | 44.44444 |
| rs12300845 | G | T | 0.058 | 0.967 | 0.007 | 4.55E-18 | 68.65306 |
| rs12516176 | C | T | 0.016 | 0.274 | 0.003 | 4.17E-09 | 28.44444 |
| rs12540285 | A | G | 0.02 | 0.78 | 0.003 | 6.43E-12 | 44.44444 |
| rs12620844 | C | T | 0.014 | 0.431 | 0.002 | 1.08E-08 | 49 |
| rs12941913 | T | C | 0.016 | 0.608 | 0.002 | 2.12E-10 | 64 |
| rs12992747 | A | C | 0.016 | 0.228 | 0.003 | 1.66E-08 | 28.44444 |
| rs13013 | A | C | 0.015 | 0.584 | 0.002 | 9.15E-10 | 56.25 |
| rs1348675 | A | G | 0.019 | 0.233 | 0.003 | 2.12E-11 | 40.11111 |
| rs141179989 | C | T | 0.055 | 0.983 | 0.01 | 3.13E-08 | 30.25 |
| rs1441171 | G | T | 0.023 | 0.476 | 0.002 | 5.17E-21 | 132.25 |
| rs1476698 | G | A | 0.018 | 0.369 | 0.002 | 1.28E-12 | 81 |
| rs149624078 | C | T | 0.13 | 0.986 | 0.01 | 1.69E-35 | 169 |
| rs150844304 | C | A | 0.08 | 0.025 | 0.008 | 3.38E-25 | 100 |
| rs1545536 | C | T | 0.02 | 0.78 | 0.003 | 8.01E-12 | 44.44444 |
| rs17138478 | A | C | 0.034 | 0.129 | 0.004 | 5.62E-22 | 72.25 |
| rs172305 | G | A | 0.014 | 0.687 | 0.003 | 3.91E-08 | 21.77778 |
| rs17308476 | T | C | 0.022 | 0.111 | 0.004 | 7.11E-09 | 30.25 |
| rs17652767 | G | A | 0.027 | 0.894 | 0.004 | 3.48E-12 | 45.5625 |
| rs17781691 | A | G | 0.017 | 0.506 | 0.002 | 4.56E-12 | 72.25 |
| rs1800693 | T | C | 0.02 | 0.599 | 0.002 | 7.10E-16 | 100 |
| rs1800973 | A | C | 0.03 | 0.061 | 0.005 | 1.85E-09 | 36 |
| rs1859726 | C | T | 0.023 | 0.093 | 0.004 | 1.66E-08 | 33.0625 |
| rs1889316 | C | T | 0.056 | 0.846 | 0.003 | 2.60E-63 | 348.4444 |
| rs1933736 | C | T | 0.019 | 0.401 | 0.002 | 6.28E-15 | 90.25 |
| rs1985157 | C | T | 0.016 | 0.413 | 0.002 | 1.68E-11 | 64 |
| rs2011689 | A | G | 0.013 | 0.389 | 0.002 | 4.06E-08 | 42.25 |

| | | | | | | | |
|---|---|---|---|---|---|---|---|
| rs204914 | T | C | 0.059 | 0.047 | 0.006 | 3.07E-25 | 96.69444 |
| rs2110944 | C | T | 0.016 | 0.531 | 0.002 | 5.91E-11 | 64 |
| rs2161037 | A | G | 0.022 | 0.545 | 0.002 | 2.32E-19 | 121 |
| rs2161374 | C | T | 0.017 | 0.513 | 0.002 | 4.28E-12 | 72.25 |
| rs2239222 | G | A | 0.034 | 0.349 | 0.003 | 4.78E-42 | 128.4444 |
| rs2246833 | T | C | 0.026 | 0.34 | 0.003 | 1.65E-25 | 75.11111 |
| rs2250010 | T | C | 0.022 | 0.81 | 0.003 | 2.72E-13 | 53.77778 |
| rs2393794 | C | T | 0.036 | 0.186 | 0.003 | 3.90E-31 | 144 |
| rs2432195 | T | C | 0.018 | 0.827 | 0.003 | 9.68E-09 | 36 |
| rs2700938 | C | T | 0.022 | 0.376 | 0.002 | 3.78E-19 | 121 |
| rs2836881 | G | T | 0.032 | 0.733 | 0.003 | 8.98E-32 | 113.7778 |
| rs2847289 | C | A | 0.022 | 0.572 | 0.002 | 3.36E-20 | 121 |
| rs28929474 | C | T | 0.1 | 0.98 | 0.008 | 2.19E-32 | 156.25 |
| rs3125326 | C | A | 0.014 | 0.609 | 0.002 | 1.45E-08 | 49 |
| rs340023 | T | C | 0.022 | 0.269 | 0.003 | 8.45E-16 | 53.77778 |
| rs34139656 | G | A | 0.02 | 0.328 | 0.003 | 1.39E-14 | 44.44444 |
| rs34298354 | C | T | 0.027 | 0.878 | 0.004 | 1.97E-13 | 45.5625 |
| rs34761529 | C | T | 0.017 | 0.795 | 0.003 | 2.20E-08 | 32.11111 |
| rs3746778 | G | A | 0.015 | 0.58 | 0.002 | 1.03E-09 | 56.25 |
| rs4006577 | G | A | 0.014 | 0.379 | 0.002 | 6.54E-09 | 49 |
| rs4148155 | A | G | 0.021 | 0.886 | 0.004 | 1.42E-08 | 27.5625 |
| rs4655802 | G | A | 0.023 | 0.41 | 0.002 | 6.11E-22 | 132.25 |
| rs4656849 | G | A | 0.046 | 0.601 | 0.002 | 8.39E-79 | 529 |
| rs469802 | A | G | 0.035 | 0.796 | 0.003 | 1.04E-31 | 136.1111 |
| rs4704093 | G | T | 0.017 | 0.468 | 0.002 | 1.27E-12 | 72.25 |
| rs4764939 | C | T | 0.018 | 0.531 | 0.002 | 9.33E-16 | 81 |
| rs56015600 | G | A | 0.04 | 0.63 | 0.002 | 1.48E-58 | 400 |
| rs6012927 | G | A | 0.016 | 0.358 | 0.003 | 6.05E-10 | 28.44444 |
| rs60987662 | G | A | 0.015 | 0.38 | 0.002 | 3.52E-10 | 56.25 |
| rs61542988 | C | T | 0.026 | 0.754 | 0.003 | 1.99E-21 | 75.11111 |
| rs62618693 | C | T | 0.031 | 0.955 | 0.006 | 3.94E-08 | 26.69444 |
| rs6920220 | A | G | 0.021 | 0.223 | 0.003 | 9.11E-14 | 49 |
| rs704017 | A | G | 0.019 | 0.428 | 0.002 | 1.36E-14 | 90.25 |
| rs7084062 | G | A | 0.018 | 0.489 | 0.002 | 8.85E-14 | 81 |
| rs714052 | A | G | 0.031 | 0.875 | 0.004 | 3.38E-18 | 60.0625 |
| rs7250946 | T | C | 0.034 | 0.044 | 0.006 | 7.65E-09 | 32.11111 |
| rs7317323 | C | T | 0.029 | 0.057 | 0.005 | 1.52E-08 | 33.64 |
| rs75460349 | A | C | 0.091 | 0.976 | 0.008 | 3.77E-30 | 129.3906 |
| rs762360 | C | T | 0.017 | 0.301 | 0.003 | 2.49E-10 | 32.11111 |
| rs77704739 | T | C | 0.051 | 0.958 | 0.006 | 6.65E-18 | 72.25 |
| rs7828742 | G | A | 0.026 | 0.598 | 0.002 | 5.94E-27 | 169 |
| rs7833554 | T | C | 0.014 | 0.555 | 0.002 | 7.26E-09 | 49 |
| rs7846549 | G | A | 0.035 | 0.896 | 0.004 | 4.09E-19 | 76.5625 |

| | | | | | | | |
|---|---|---|---|---|---|---|---|
| rs7956514 | G | T | 0.017 | 0.287 | 0.003 | 1.16E-10 | 32.11111 |
| rs799260 | G | A | 0.023 | 0.834 | 0.003 | 9.08E-13 | 58.77778 |
| rs80292319 | T | C | 0.028 | 0.941 | 0.005 | 3.03E-08 | 31.36 |
| rs8060025 | T | G | 0.019 | 0.389 | 0.002 | 3.87E-14 | 90.25 |
| rs8178824 | T | C | 0.06 | 0.03 | 0.007 | 1.31E-17 | 73.46939 |
| rs889745 | T | G | 0.014 | 0.524 | 0.002 | 1.67E-08 | 49 |
| rs9604045 | G | T | 0.023 | 0.75 | 0.003 | 1.36E-15 | 58.77778 |
| rs9638882 | C | A | 0.024 | 0.791 | 0.003 | 1.24E-16 | 64 |
| rs9738365 | A | C | 0.02 | 0.267 | 0.003 | 5.94E-14 | 44.44444 |
| rs9838974 | A | G | 0.014 | 0.41 | 0.002 | 9.49E-09 | 49 |

Note: This table lists the single nucleotide polymorphisms (SNPs) used as instrumental variables for C-reactive protein (CRP) in the Mendelian Randomization analysis. The summary statistics for the association of each SNP with CRP were obtained from the UK Biobank GWAS. The effect allele is the CRP-increasing allele, and the beta represents the per-allele increase in standardized, log-transformed CRP levels. The F-statistic, a measure of instrument strength, was calculated as F=(beta/se)2. Abbreviations: SNP, single nucleotide polymorphism; SE, standard error; EAF, effect allele frequency.

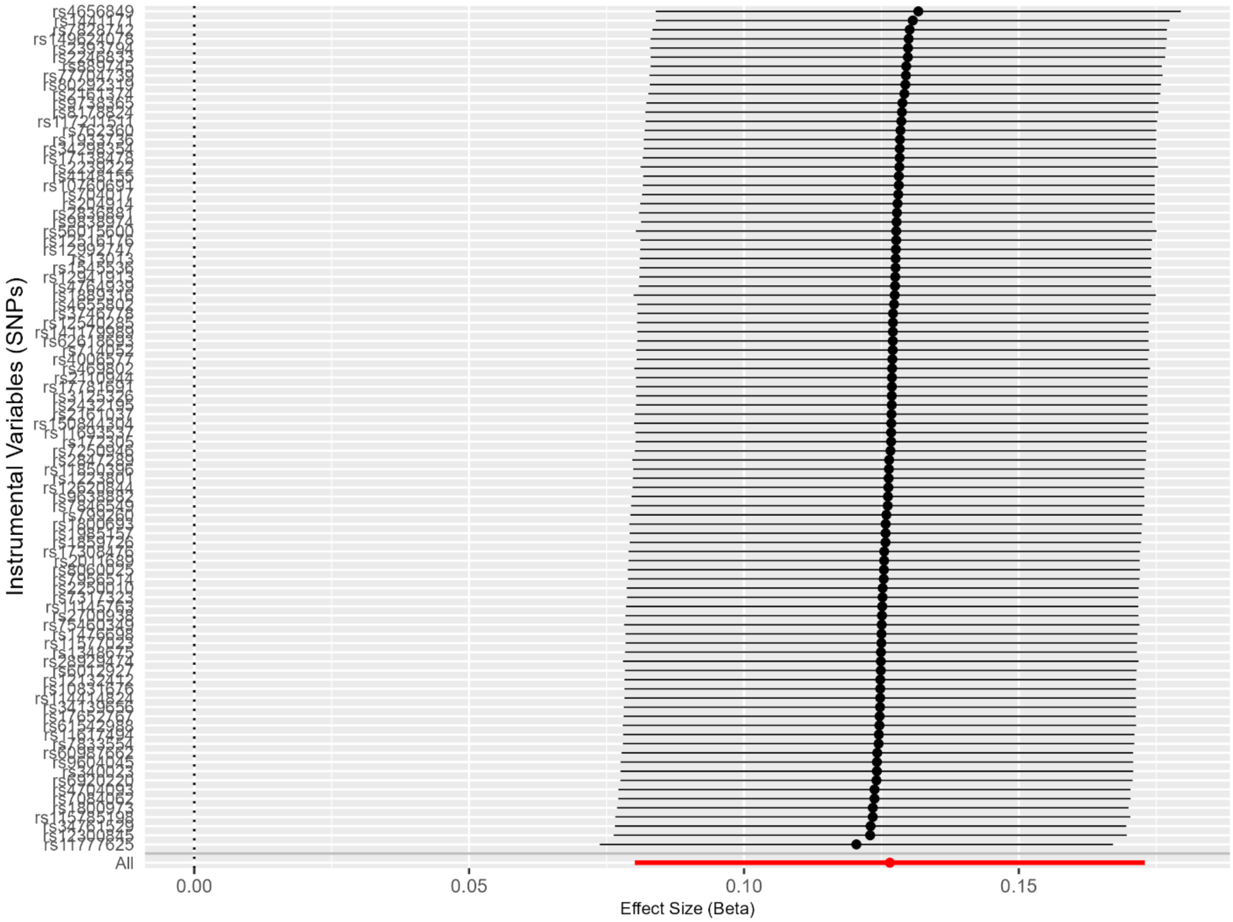


eFigure 1: Leave-one-out Analysis

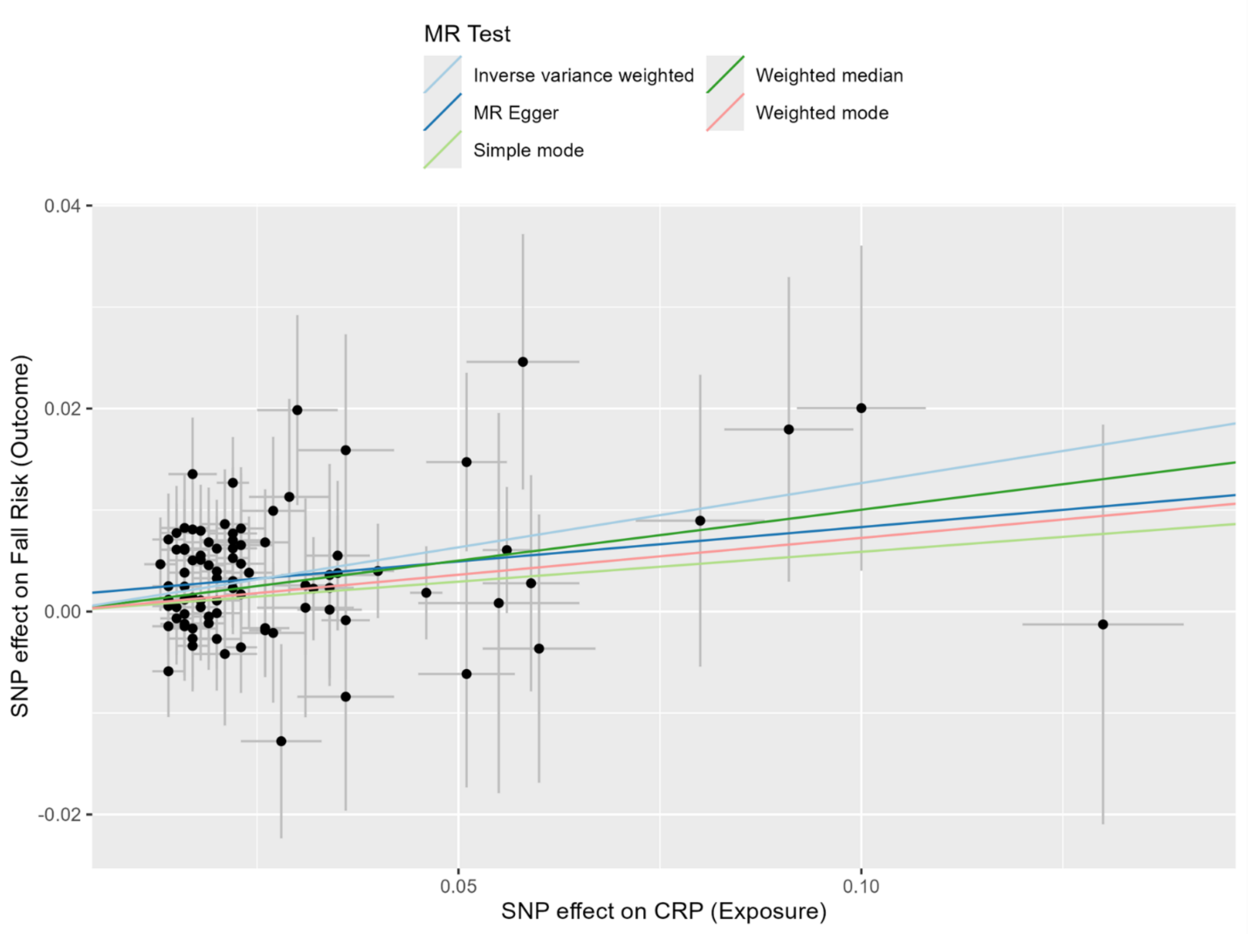


eFigure 2: Scatter plot of the SNP-CRP associations against SNP-fall risk associations

**STROBE-MR checklist of recommended items to address in reports of Mendelian randomization studies**[1 2]

| Item No. | Section | Checklist item | Page No. | Relevant text from manuscript |
|---|---|---|---|---|
| 1 | **TITLE and ABSTRACT** | Indicate Mendelian randomization (MR) as the study's design in the title and/or the abstract if that is a main purpose of the study | 1, 3 | (1) Title: Association of Systemic Inflammation with Balance and Falls in Older Adults: NHANES and Mendelian Randomization Study<br>(2) This study investigates the association between CRP levels and balance using observational data and Mendelian Randomization (MR) to explore its causal role in fall risk. |
| | **INTRODUCTION** | | | |
| 2 | **Background** | Explain the scientific background and rationale for the reported study. What is the exposure? Is a potential causal relationship between exposure and outcome plausible? Justify why MR is a helpful method to address the study question | 4, 5 | Although systemic inflammation has been linked to physical function, the specific relationship between CRP levels and balance performance remains inadequately explored.<br>Mendelian Randomization (MR) provides a powerful tool to clarify this uncertainty. By using genetic variants associated with CRP levels as instrumental variables (IVs), MR analysis can infer causal relationships while reducing bias from confounding and reverse causation. Recent genome-wide association studies (GWAS) have identified strong genetic instruments for CRP, enabling the application of MR methodologies. Additionally, GWAS datasets on fall risk, balance performance, and mobility offer a valuable opportunity to investigate the causal role of systemic inflammation in fall susceptibility.<br>This study combines observational and genetic approaches to provide a comprehensive analysis of the relationship between CRP and balance performance. |
| 3 | **Objectives** | State specific objectives clearly, including pre-specified causal hypotheses (if any). State that MR is a method that, under specific assumptions, intends to estimate causal effects | 5 | By combining NHANES observational data with MR analysis, this study aims to clarify the role of systemic inflammation in balance performance and its implications for fall risk. Should a causal link between CRP levels and balance dysfunction be confirmed, it could guide the development of targeted anti-inflammatory interventions to enhance balance and reduce fall risk in aging populations. |
| | **METHODS** | | | |
| 4 | **Study design and data sources** | Present key elements of the study design early in the article. Consider including a table listing sources of data for all phases of the study. For each data source contributing to the analysis, describe the following: | | |

| | | | | | |
|---|---|---|---|---|---|
| | | a) | Setting: Describe the study design and the underlying population, if possible. Describe the setting, locations, and relevant dates, including periods of recruitment, exposure, follow-up, and data collection, when available. | 6 | |
| | | b) | Participants: Give the eligibility criteria, and the sources and methods of selection of participants. Report the sample size, and whether any power or sample size calculations were carried out prior to the main analysis | 6 | For this study, we analyzed cross-sectional data from the NHANES 2021–2023 cycle, which included 8,727 participants with CRP measurements and 4,771 participants with balance assessments. To focus on older adults, we restricted the analysis to individuals aged ≥60 years. This threshold is consistent with the definition of an older person used by the United Nations and other aging research. Participants with missing serum CRP levels and/or balance performance data were excluded, yielding a final study sample of 1,215 participants. |
| | | c) | Describe measurement, quality control and selection of genetic variants | 7, 8, 10 | 1.2. C-Reactive Protein<br>1.3. Assessment of Balance<br>2.2 Mendelian Randomization Study<br>2.2.1 Selection of Genetic Instruments |
| | | d) | For each exposure, outcome, and other relevant variables, describe methods of assessment and diagnostic criteria for diseases | 7, 8 | 1.2. C-Reactive Protein<br>1.3. Assessment of Balance |
| | | e) | Provide details of ethics committee approval and participant informed consent, if relevant | 6 | The NCHS Ethics Review Committee approved NHANES, and all participants provided written informed consent before enrollment. The NCHS Research Ethics Review Board authorized the study under protocol #2021-05. NHANES follows strict protocols to ensure confidentiality and protect against identification. Since this analysis utilized publicly available data, additional institutional review board approval was not required. |
| 5 | **Assumptions** | | Explicitly state the three core IV assumptions for the main analysis (relevance, independence and exclusion restriction) as well assumptions for any additional or sensitivity analysis | | |
| 6 | **Statistical methods: main analysis** | | Describe statistical methods and statistics used | | |
| | | a) | Describe how quantitative variables were handled in the analyses (i.e., scale, units, model) | 8, 9 | 2.1. Ordinal Logistic Regression |
| | | b) | Describe how genetic variants were handled in the analyses and, if applicable, how their weights were selected | 10, 11 | 2.2.1 Selection of Genetic Instruments |

| | | | | | |
|---|---|---|---|---|---|
| | | c) | Describe the MR estimator (e.g. two-stage least squares, Wald ratio) and related statistics. Detail the included covariates and, in case of two-sample MR, whether the same covariate set was used for adjustment in the two samples | 11, 12 | 2.2.2. MR Analysis |
| | | d) | Explain how missing data were addressed | 6, 8 | Participants with missing serum CRP levels and/or balance performance data were excluded, yielding a final study sample of 1,215 participants.<br>Missing data for the covariates were imputed with the mice package, and the imputed dataset was used for all analyses. |
| | | e) | If applicable, indicate how multiple testing was addressed | | |
| 7 | **Assessment of assumptions** | | Describe any methods or prior knowledge used to assess the assumptions or justify their validity | | |
| 8 | **Sensitivity analyses and additional analyses** | | Describe any sensitivity analyses or additional analyses performed (e.g. comparison of effect estimates from different approaches, independent replication, bias analytic techniques, validation of instruments, simulations) | | |
| 9 | **Software and pre-registration** | | | | |
| | | a) | Name statistical software and package(s), including version and settings used | 12 | All analyses were performed using the TwoSampleMR package and the MR-PRESSO framework in R software (version 4.2.3). |
| | | b) | State whether the study protocol and details were pre-registered (as well as when and where) | | |
| | **RESULTS** | | | | |
| 10 | **Descriptive data** | | | | |
| | | a) | Report the numbers of individuals at each stage of included studies and reasons for exclusion. Consider use of a flow diagram | | Figure 1: Inclusion and exclusion criteria |
| | | b) | Report summary statistics for phenotypic exposure(s), outcome(s), and other relevant variables (e.g. means, SDs, proportions) | | Table 1: Baseline Characteristics of the Study Population by Gender |
| | | c) | If the data sources include meta-analyses of previous studies, provide the assessments of heterogeneity across these studies | | NA |

| | | | | | |
|---|---|---|---|---|---|
| | | d) | For two-sample MR:<br>i. Provide justification of the similarity of the genetic variant-exposure associations between the exposure and outcome samples<br>ii. Provide information on the number of individuals who overlap between the exposure and outcome studies | | |
| 11 | **Main results** | | | | |
| | | a) | Report the associations between genetic variant and exposure, and between genetic variant and outcome, preferably on an interpretable scale | 13 | The primary MR analysis performed using the IVW method (Table 3), demonstrated a significant association between CRP levels and fall risk (OR=1.13, 95% CI: 1.08–1.19, p <.001). |
| | | b) | Report MR estimates of the relationship between exposure and outcome, and the measures of uncertainty from the MR analysis, on an interpretable scale, such as odds ratio or relative risk per SD difference | 13 | The Weighted Median method produced consistent results (OR=1.11, 95% CI: 1.04–1.18, p=.003). However, both the Weighted Mode (OR=1.08, 95% CI: 0.96–1.21, p=.217) and Simple Mode (OR=1.06, 95% CI: .91–1.23, p=.449) methods did not reveal significant associations. The MR-Egger method also produced a non-significant causal estimate (OR=1.07, 95% CI: .96–1.19, p=.229), with its intercept showing no evidence of directional pleiotropy (p=.250). |
| | | c) | If relevant, consider translating estimates of relative risk into absolute risk for a meaningful time period | | |
| | | d) | Consider plots to visualize results (e.g. forest plot, scatterplot of associations between genetic variants and outcome versus between genetic variants and exposure) | | |
| 12 | **Assessment of assumptions** | | | | |
| | | a) | Report the assessment of the validity of the assumptions | | |
| | | b) | Report any additional statistics (e.g., assessments of heterogeneity across genetic variants, such as $I^2$, Q statistic or E-value) | 13 | Cochran's Q statistic indicated no significant heterogeneity among the genetic instruments for both the IVW method (Q=63.44, p=.988) and the MR-Egger method (Q=62.10, p=.989). |
| 13 | **Sensitivity analyses and additional analyses** | | | | |
| | | a) | Report any sensitivity analyses to assess the robustness of the main results to violations of the assumptions | 13, 14 | Finally, a leave-one-out sensitivity analysis confirmed the robustness of our findings, as the exclusion of any single SNP did not substantially affect the results (eFigure 1). |

| | | | | |
|---|---|---|---|---|
| | | b) Report results from other sensitivity analyses or additional analyses | 13 | Additionally, the MR-PRESSO global test detected no significant outliers (p=.990). |
| | | c) Report any assessment of direction of causal relationship (e.g., bidirectional MR) | | |
| | | d) When relevant, report and compare with estimates from non-MR analyses | | |
| | | e) Consider additional plots to visualize results (e.g., leave-one-out analyses) | | eFigure 1: Plot of Leave-one-out Sensitivity Analysis Results |
| | **DISCUSSION** | | | |
| 14 | **Key results** | Summarize key results with reference to study objectives | 14 | Our analysis of the NHANES data revealed a significant link between elevated CRP levels and poor balance performance in the overall sample, with a particularly strong association observed in males. This relationship remained significant across increasingly adjusted models, supporting its robustness and independence from demographic, socioeconomic, health, and lifestyle factors. However, no significant association was found in females, suggesting potential gender differences in the effects of systemic inflammation on balance. |
| 15 | **Limitations** | Discuss limitations of the study, taking into account the validity of the IV assumptions, other sources of potential bias, and imprecision. Discuss both direction and magnitude of any potential bias and any efforts to address them | 16, 17 | Our study has several limitations. First, the generalizability of our findings may be constrained. Although NHANES represents the U.S. population, the genetic data used in our MR analysis is derived primarily from individuals of European ancestry. As a result, these findings may not fully apply to populations with different genetic backgrounds, lifestyles, or healthcare systems. Additionally, the NHANES dataset's diverse, multiracial composition may introduce variability in the observational results, as genetic predispositions and environmental factors can influence balance performance.<br><br>Another limitation is the cross-sectional design of the NHANES data, which prevents us from establishing causal or temporal relationships between CRP levels and balance performance. Moreover, while the MRT used to assess balance performance is practical, we acknowledge that balance impairment and fall risk are related but distinct constructs. The ordinal MRT score models gradations in postural control, but does not directly quantify fall events. Despite adjusting for numerous potential confounders, unmeasured factors, such as diet, medication use, other inflammatory markers, or muscle strength, may still influence the observed associations. Notably, grip strength or other direct measures of muscular performance were not available in the 2021–2023 NHANES cycle, limiting our ability to account for the influence of muscular strength on balance. Additionally, the single-time point CRP measurements in NHANES do not capture potential short-term fluctuations or the effects of chronic inflammation over time. Finally, our study focused exclusively on CRP as a biomarker of systemic inflammation. While CRP is a robust and clinically relevant marker, the inclusion of other pro- |

| | | | | | |
|---|---|---|---|---|---|
| | | | | | inflammatory cytokines, such as Interleukin-6 or Tumor Necrosis Factor-alpha, would have provided a more complete assessment of the inflammatory pathways involved. The unavailability of these markers in the recent NHANES public-use dataset precluded such an analysis. |
| 16 | **Interpretation** | | | | |
| | | a) | Meaning: Give a cautious overall interpretation of results in the context of their limitations and in comparison with other studies | 15 | This study adds to the growing evidence linking systemic inflammation to physical function and balance performance. Previous research has highlighted the detrimental effects of elevated CRP levels on various aspects of physical performance, including gait speed, grip strength, and frailty. Our NHANES analysis supports these findings, showing a strong association between CRP levels and impaired balance performance, especially in males, across multiple models that accounted for demographic, socioeconomic, health, and lifestyle factors. |
| | | b) | Mechanism: Discuss underlying biological mechanisms that could drive a potential causal relationship between the investigated exposure and the outcome, and whether the gene-environment equivalence assumption is reasonable. Use causal language carefully, clarifying that IV estimates may provide causal effects only under certain assumptions | | |
| | | c) | Clinical relevance: Discuss whether the results have clinical or public policy relevance, and to what extent they inform effect sizes of possible interventions | 17 | Targeting chronic inflammation may be a promising strategy to improve balance and reduce fall risk. Future research should aim to validate these findings and explore effective interventions. |
| 17 | **Generalizability** | | Discuss the generalizability of the study results (a) to other populations, (b) across other exposure periods/timings, and (c) across other levels of exposure | | |
| | **OTHER INFORMATION** | | | | |
| 18 | **Funding** | | Describe sources of funding and the role of funders in the present study and, if applicable, sources of funding for the databases and original study or studies on which the present study is based | 17 | This work was supported by the Science and Technology Project of Binzhou Medical University (grant no. BY2022KYQD33). |
| 19 | **Data and data sharing** | | Provide the data used to perform all analyses or report where and how the data can be accessed, and reference these sources in the article. Provide the statistical code needed to reproduce the results in the article, or report whether the code is publicly accessible and if so, where | 6, 9, 10 | We obtained cross-sectional data from the 2021–2023 cycle of the NHANES, a population-based program conducted by the National Center for Health Statistics (NCHS). The NHANES dataset is accessible through the official website (https://wwwn.cdc.gov/nchs/nhanes/continuousnhanes/default.aspx?Cycle=2021-2023). |

| | | | | |
|---|---|---|---|---|
| | | | | We extracted genome-wide significant SNPs (p<.001) associated with serum CRP levels from the UK Biobank summary statistics via the IEU OpenGWAS database (https://gwas.mrcieu.ac.uk/datasets/ukb-d-30710_irnt/).<br>For fall risk, we sourced GWAS summary-level data from the GWAS Catalog (https://www.ebi.ac.uk/gwas/rest/api/studies/GCST90012857), which included 2,215 cases and 6,289 controls, all of European ancestry. |
| 20 | **Conflicts of Interest** | All authors should declare all potential conflicts of interest | 17 | The authors report no conflicts of interest. |

This checklist is copyrighted by the Equator Network under the Creative Commons Attribution 3.0 Unported (CC BY 3.0) license.

1. Skrivankova VW, Richmond RC, Woolf BAR, Yarmolinsky J, Davies NM, Swanson SA, et al. Strengthening the Reporting of Observational Studies in Epidemiology using Mendelian Randomization (STROBE-MR) Statement. JAMA. 2021;under review.

2. Skrivankova VW, Richmond RC, Woolf BAR, Davies NM, Swanson SA, VanderWeele TJ, et al. Strengthening the Reporting of Observational Studies in Epidemiology using Mendelian Randomisation (STROBE-MR): Explanation and Elaboration. BMJ. 2021;375:n2233.
